# Genetic Variants in the Fat and Obesity Associated (FTO) Gene and Risk of Alzheimer's Disease

**DOI:** 10.1371/journal.pone.0050354

**Published:** 2012-12-12

**Authors:** Christiane Reitz, Giuseppe Tosto, Richard Mayeux, Jose A. Luchsinger, the NIA-LOAD/NCRAD Family Study Group and the Alzheimer's Disease Neuroimaging Initiative

**Affiliations:** 1 The Taub Institute for Research on Alzheimer's Disease and the Aging Brain, College of Physicians and Surgeons, Columbia University, New York, New York, United States of America; 2 The Gertrude H. Sergievsky Center, College of Physicians and Surgeons, Columbia University, New York, New York, United States of America; 3 Department of Neurology, College of Physicians and Surgeons, Columbia University, New York, New York, United States of America; 4 Department of Psychiatry, College of Physicians and Surgeons, Columbia University, New York, New York, United States of America; 5 Department of Medicine, Pathology, College of Physicians and Surgeons, Columbia University, New York, New York, United States of America; University of Kentucky, United States of America

## Abstract

**Background:**

Recent studies showed that polymorphisms in the Fat and Obesity-Associated *(FTO)* gene have robust effects on obesity, obesity-related traits and endophenotypes associated with Alzheimer's disease (AD).

**Methods:**

We used 1,877 Caucasian cases and controls from the NIA-LOAD study and 1,093 Caribbean Hispanics to further explore the association of *FTO* with AD. Using logistic regression, we assessed 42 SNPs in introns 1 and 2, the region previously reported to be associated with AD endophenotypes, which had been derived by genome-wide screenings. In addition, we performed gene expression analyses of neuropathologically confirmed AD cases and controls of two independent datasets (19 AD cases, 10 controls; 176 AD cases, 188 controls) using within- and between-group factors ANOVA of log_10_ transformed rank invariant normalized expression data.

**Results:**

In the NIALOAD study, one SNP was significantly associated with AD and three additional markers were close to significance (rs6499640, rs10852521, rs16945088, rs8044769, FDR p-value: 0.05<p<0.09). Two of the SNPs are in strong LD (D′>0.9) with the previously reported SNPs. In the Caribbean Hispanic dataset, we identified three SNPs (rs17219084, rs11075996, rs11075997, FDR p-value: 0.009<p<0.01) that were associated with AD. These results were confirmed by haplotype analyses and in a metaanalysis in which we included the ADNI dataset. *FTO* had a significantly lower expresssion in AD cases compared to controls in two independent datasets derived from human cortex and amygdala tissue, respectively (p = 2.18×10−5 and p<0.0001).

**Conclusions:**

Our data support the notion that genetic variation in Introns 1 and 2 of the FTO gene may contribute to AD risk.

## Introduction

Alzheimer's disease (AD) is the most common cause of dementia, accounting for 60–80% of cases [1]. At present, about 33.9 million people worldwide have AD, and the prevalence is anticipated to triple over the next 40 years owing to demographic changes and longer life expectancies [1]. Available drugs for dementia and AD have small effect sizes and do not clearly alter disease progression [2].

As delaying symptom onset by as little as 1 year could potentially lower AD prevalence by more than 9 million cases over the next 40 years [1], there has been growing interest in identification of preventive measures. Observational studies have assessed a wide range of potentially modifiable risk factors, in particular cardiovascular risk factors. While for diabetes the association with AD seems clear [3], [4], the association for most other cardiovascular risk factors, including obesity, remains largely inconsistent across studies. For obesity, most studies show an increased risk [5], but some show an inverse risk [6], [7], some show nonlinear associations [8], and some show no association [9]. Explanations for the conflicting data include reversed causation, residual confounding, potential survival bias, and decreased validity of body mass index (BMI) as a measure of obesity in the elderly [10]. In general, measures of central obesity, particularly waist to hip ratio (WHR), seem to be better predictors of cardiovascular outcomes compared with BMI [11], and central obesity in middle age is related to a higher risk of dementia.

**Figure 1:**
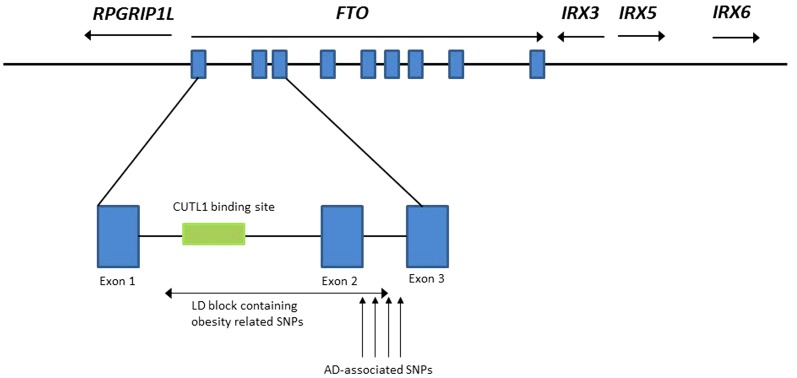
Genomic organization of *FTO* and its neighboring genes (not drawn to scale). The *FTO* gene contains nine exons which are depicted in blue rectangles. The SNPs previously reported to be associated with obesity-related measures or AD endophenotypes, as well as the SNPs associated with AD in the present study, are located in Intron 1, Exon 2 and Intron 2.

Recent studies have demonstrated that polymorphisms in the Fat and Obesity-Associated (FTO) gene have strong and robust effects on obesity and obesity-related traits (such as body mass index (BMI), waist circumference, waist to hip ratio, bicondilar upper arm width and upper arm circumference) [12], [13], [14], [15]. *FTO* is located on chromosome 16q12.2, has nine known splice variants and is highly expressed in the brain. Although this gene has nine exons, all reported polymorphisms are part of one LD block spanning 47 kb across intron 1, exon 2 and part of intron 2 (Figure 1).

The same polymorphisms have also independent strong effects on insulin resistance/Type 2 Diabetes, which is – as described above- a strong risk factor for AD [12], [13], [14], metabolic syndrome [16], obesity-related dyslipidemia [17], and changes in blood pressure [18]. In addition, several studies reported associations of genetic variation in *FTO* with traits that are common endophenotypes of dementia. In the Alzheimer's Disease Neuroimaging Initiative (ADNI), the *FTO* polymorphism most commonly associated with obesity and in Caucasians (rs9939609 (Intron 1)) was associated with reductions in frontal and occipital lobe volumes [19]. In a Swedish dataset involving 355 old men at the age of 82 years from the Uppsala Longitudinal Study of Adult Men (ULSAM), rs9939609 was associated with impairment in verbal fluency [20]. In the only study to date that assessed the effect of genetic variation in *FTO* on AD risk, a longitudinal cohort study of the Kungsholmen project that involved 1,003 Caucasians followed for 9 years, the minor allele of rs9939609 was associated with a 1.6-fold risk of developing AD [21]. The advantage of relating genetic variation with a phenotype of interest is that it overcomes the issues of reverse causation and residual confounding [22].

The goal of the present study was to further clarify whether genetic variation in *FTO*, that is similar to or in linkage disequilibrium (LD) with the SNPs previously reported to be associated with obesity-related measures or AD endophenotypes is associated with AD. We explored this question by genetic association analyses of two independent case-control datasets that are derived from different ethnic groups and have sufficient power to detect modest effect sizes. In addition, we peformed a meta-analysis that also included the publicly available ADNI dataset, and conducted microarray gene expression analyses of two independent samples.

## Methods

### Participants

The two datasets used for the discovery single marker analyses included (1) 1,877 cases and controls from the NIA-LOAD study [23], and (2) 1,093 cases and controls from a Caribbean Hispanic dataset [24].

For the NIALOAD study, recruitment took place throughout the United States at 18 participating AD centers (ADCs), each of which had received approval by their institutional review board. A collaborative effort by each ADC, the NIA, the Alzheimer's Disease Education and Referral Center, and the Alzheimer's Association led to national media coverage, which facilitated recruitment. A toll-free number at the National Cell Repository for Alzheimer's Disease (http://ncrad.iu.edu) was made available. When qualifying families contacted the National Cell Repository, research staff referred the family to the geographically closest participating ADC for evaluation. The recruitment criteria included a family with multiple members affected with LOAD that could provide clinical information and a biological sample for DNA extraction. The proband had to have a diagnosis of definite or probable LOAD [25] with onset after 60 years of age and a full sibling with definite, probable, or possible LOAD with onset after 60 years of age. A third biologically related family member was required, who could have been a first-, second-, or third-degree relative of the affected sibling pairs and 60 years or older if unaffected or 50 years or older if diagnosed as having LOAD or mild cognitive impairment [26]. Unaffected persons were required to have had documented cognitive testing and clinical examination results to verify the clinical designation. A minimal data set included demographic variables, diagnosis, age at onset, method of diagnosis, Clinical Dementia Rating Scale score [27], and the presence of other relevant health problems. Each ADC was required to use standard research criteria for the diagnosis of LOAD [25]. Participants with advanced disease or those living in a remote location who could not complete a detailed in-person evaluation contributed blood samples, and the site investigator conducted a detailed review of medical records to document the presence or absence of LOAD.

The 1,093 Caribbean Hispanic subjects were selected from the Washington Heights–Inwood Columbia Aging Project (WHICAP) study and the Estudio Familiar de Influencia Genetica de Alzheimer (EFIGA) study. The WHICAP study [28] is a population-based epidemiologic study of randomly selected elderly individuals residing in northern Manhattan, New York, comprising three ethnic groups: non-Hispanic white, Caribbean Hispanic, and African American. For the current study, only individuals who were self-reported Hispanic of Caribbean origin were included. In addition, we selected one affected individual from each family participating in the EFIGA study of Caribbean Hispanic families with LOAD [29]. Both studies followed the same clinical diagnostic methods. The participants originated from the Dominican Republic and Puerto Rico. Approximately 60.3% of the affected individuals were participants in the WHICAP epidemiologic study, and the remaining 39.7% of the participants were from the EFIGA study. All unaffected individuals were participants in the WHICAP epidemiologic study. For the familial cases, we selected one proband from each family to create a cohort of unrelated individuals. We selected persons with definite or probable LOAD over those with possible LOAD to limit the effects of comorbidity. Data were available from medical, neurological, and neuropsychological evaluations [30] collected from 1999 through 2007. The standardized neuropsychological test battery covered multiple domains and included the Mini-Mental State Examination [31], the Boston Naming Test [32], the Controlled Word Association Test from the Boston Diagnostic Aphasia Evaluation [33], the Wechsler Adult Intelligence Scale–Revised similarities subtest [34], the Mattis Dementia Rating Scale [35], the Rosen Drawing Test [36], the Benton Visual Retention Test [37], the multiple-choice version of the Benton Visual Retention Test [37], and the Selective Reminding Test [38]. The diagnosis of dementia was established on the basis of all available information gathered from the initial and follow-up assessments and medical records. The diagnosis of LOAD was based on the National Institute of Neurological Disorders and Stroke–Alzheimer's Disease and Related Disorders Association criteria [25].

The clinical characteristics of these two datasets are summarized in Table 1. As described above, for both datasets, the diagnoses of ‘probable’ or ‘possible’ AD were defined based on the National Institute of Neurological and Communication Disorders and Stroke–Alzheimer's Disease and Related Disorders Association (NINCDS-ADRDA) diagnosis criteria at clinics specializing in memory disorders or in clinical investigations. Although both datasets were subsets of larger family samples, all samples used in the present study were unrelated. From each family, one affected individual with definite or probable LOAD was selected, and unrelated, unaffected individuals served as controls. Persons were classified as “controls” when they were without cognitive impairment or dementia at last visit [23], [24]. Informed consent was obtained in written form from all participants using procedures approved by institutional review boards at each of the clinical research centers collecting human subjects. Whether the participants had the capacity to consent was assessed by in-person interview of the participant and/or next of kin, carers or guardians. Next of kin, carers or guardians consented on the behalf of participants whose capacity to consent was reduced. Recruitment for the Caribbean Hispanic Study was approved by the Institutional Review Board of the Columbia University Medical Center. Recruitment for the NIALOAD Study was approved by the relevant institutional review boards of the participating centers (ie. the IRBs of Boston University, Columbia University, Duke University, Indiana University, Massachusetts General Hospital, Mayo Clinic, Mount Sinai School of Medicine, Oregon Health & Science University, Rush University Medical Center, University of Alabama at Birmingham, University of California Los Angeles; University of Kentucky; University of Pennsylvania; University of Pittsburgh; University of Southern California; University of Texas Southwestern; University of Washington; Washington University Medical Center; University of Miami; Northwestern University; Emory University).The study was conducted according to the principles expressed in the Declaration of Helsinki.

**Table 1:** Characteristics of the study samples.

Characteristics	NIA-LOAD (n = 1,877)	Caribbean Hispanics (n = 1,093)
Affected with AD	993	549
Unaffected	884	544
Age		
Onset: affecteds	71.6±6.9	79.9±8.0
Age at last exam: unaffecteds	76.1±8.4	78.8±6.4
Proportion of females (%)	62.3%	69.7
APOE allele frequency (%)		
e4	31.2	18.2
e3	63.3	75.1
e2	5.5	6.8

The publicly available ADNI data used in the preparation of this article were obtained from the Alzheimer's Disease Neuroimaging Initiative (ADNI) database (adni.loni.ucla.edu). The ADNI was launched in 2003 by the National Institute on Aging (NIA), the National Institute of Biomedical Imaging and Bioengineering (NIBIB), the Food and Drug Administration (FDA), private pharmaceutical companies and non-profit organizations, as a $60 million, 5-year public-private partnership. The primary goal of ADNI has been to test whether serial magnetic resonance imaging (MRI), positron emission tomography (PET), other biological markers, and clinical and neuropsychological assessment can be combined to measure the progression of mild cognitive impairment (MCI) and early Alzheimer's disease (AD). Determination of sensitive and specific markers of very early AD progression is intended to aid researchers and clinicians to develop new treatments and monitor their effectiveness, as well as lessen the time and cost of clinical trials. The Principal Investigator of this initiative is Michael W. Weiner, MD, VA Medical Center and University of California – San Francisco. ADNI is the result of efforts of many coinvestigators from a broad range of academic institutions and private corporations, and subjects have been recruited from over 50 sites across the U.S. and Canada. The initial goal of ADNI was to recruit 800 adults, ages 55 to 90, to participate in the research, approximately 200 cognitively normal older individuals to be followed for 3 years, 400 people with MCI to be followed for 3 years and 200 people with early AD to be followed for 2 years. Also this study complied with the Declaration of Helsinki.

### Genotyping

For both studies, we used the results from direct genotyping of single nucleotide polymorphisms (SNPs) in *FTO* that was conducted as part of genome-wide studies described previously [23], [24]. For the analyses described in this study, we focused on the SNPs in Intron1, Exon 2 and Intron 2, ie. all SNPs in the regions previously reported to be associated with obesity measures, diabetes, brain volume and verbal fluency. Information on platforms used for APOE genotyping is given in Table S1.

### Microarray gene expression

For the first microarray gene expression dataset, we used brain tissue from 19 pathologically confirmed AD cases and 10 pathologically confirmed controls from the New York Brain Bank (www.nybb.hs.columbia.edu). For each of these brains, expression profiling was performed separately for RNA isolated from the cerebellum, the parietal-occipital neocortex and the amygdala. Frozen brain tissue was ground over liquid nitrogen and stored at −80°C until use. Total RNA was extracted and purified using TRIzol Plus RNA purification kit (Invitrogen). Quantification and qualification of all RNA preparations was performed using an Agilent Bioanalyzer (RNA 6000 nano-kit) and only samples with RNA integrity number (RIN)>8 were used in the subsequent RNA amplification and hybridization steps. The Genechip expression two-cycle target labeling kit (Affymetrix) was used for all samples according to Affymetrix protocols. Finally, the Affymetrix GeneChip® Human Exon 1.0 ST Arrays was used for the expression profiling. The three-region approach allowed us to enhance the signal-to-noise ratio [39], and to determine those changes in expression patterns of candidate genes that are specific for late-onset AD and consistent with distribution of AD pathology. The second gene expression dataset was a publicly available dataset consisting of expression data derived from various regions of the human cortex of 188 neuropathologically confirmed controls and 176 neuropathologically confirmed AD cases that was obtained using the Illumina HumanRefseq-8 Expression BeadChip platforms (http://labs.med.miami.edu/myers/LFuN/LFuN.html). While for the New York Brain Bank dataset exon level data were available, for the publicly available dataset only gene-level data were accessible.

### Statistical methods

We restricted the analyses to the SNPs in Intron 1, Exon 2 and Intron 2 in the *FTO* gene. First, SNP marker data were assessed for deviations from Hardy-Weinberg equilibrium (HWE) at p<0.0001 in controls. Independently for each of the case-control datasets, multivariate logistic regression analyses in PLINK (http://pngu.mgh.harvard.edu/~purcell/plink/), were used to assess genotypic and allelic associations with AD risk, first adjusting for age and sex, and then in addition adjusting for APOE-ε4. In order to account for population stratification, in the Caribbean Hispanic dataset all analyses were in addition adjusted for the first three principal components derived by EIGENSTRAT (http://genepath.med.harvard.edu/~reich/Software.htm). The False Discovery Rate (FDR) [40], which controls the expected proportion of incorrectly rejected null hypotheses (type I errors) and provides a sensible balance between the number of true and false positives [41], [42], was used to account for the error in multiple comparisons. As secondary analyses, we performed 3-SNP sliding-window haplotype analyses using the same covariates for adjustment. Finally, we obtained the publicly available data on the *FTO* gene by the ADNI study [43] and performed a meta-analysis using PLINK (http://pngu.mgh.harvard.edu/~purcell/plink/metaanal.shtml). To determine the strength of associations between the individual *FTO* SNPs and AD, we calculated a pooled OR for each marker using fixed and random effects models using PLINK. In these analyses, the individual studies were weighted in to the final statistics based on the standard errors (SE) of the individual ORs. The p values for each SNP were corrected for multiple testing using the FDR. Between-dataset heterogeneity was tested with the chi-square distributed Q statistic.

### Statistical Analysis for the gene expression data

To determine whether *FTO* expression levels differ between AD and control brains, we performed both within- and between-group factors ANOVA using PARTEK GENOMICS SUITE 6.4 (http://www.partek.com/partekgs) of log_10_ transformed Rank invariant normalized expression data. The FDR statistic was used to account for the error in multiple comparisons.

## Results

**Figure 2:**
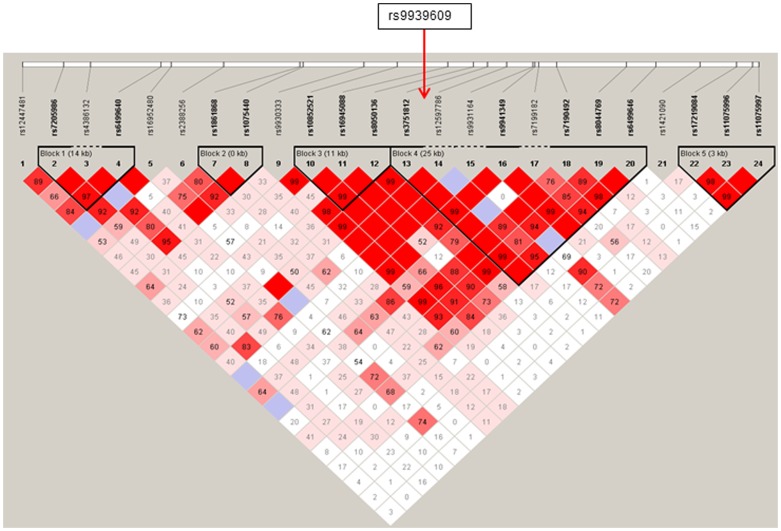
a. Linkage disequilibrium (LD) pattern in NIA-LOAD study. **b.** Linkage disequilibrium (LD) pattern in Caribbean Hispanic study.

The demographic characteristics of the NIALOAD and Caribbean Hispanic datasets are shown in table 1. In analyses of the NIALOAD study, one SNP was significantly associated with AD and three additional markers were close to significance (rs6499640, rs10852521, rs16945088, rs8044769, p-value: 0.05<p<0.09, table 2). Out of these, three markers (rs10852521, rs16945088, rs8044769) are in tight LD with the previously reported SNPs (D′>0.9; Figure 2a). In the Caribbean Hispanic dataset, we identified three SNPs (rs17219084, rs11075996, rs11075997, p-value: 0.009<p<0.01) that were significantly associated with AD. In addition, rs9931164 was close to significance (table 2). This SNP is in the same LD block as the previously reported SNPs (Figure 2b), and is independently in LD with the other four significant SNPs (Figure 2b). In haplotype analyses, several of these SNPs were also significant (table 3). In addition, the GTA haplotype at SNPs rs9931164|rs9941349|rs7199182 was significantly associated with AD in the Caribbean Hispanic dataset. rs9941349 is a proxy SNP for rs9939609 previously reported (http://www.broadinstitute.org/mpg/snap/ldsearch.php) [21]. In metaanalyses of the Caucasian NIALOAD and ADNI datasets, three SNPs (rs6499640, rs16945088, rs6499646) were significantly associated with AD (table 4). Out of these, two were in the same LD block as the previously reported SNPs. When in addition the Caribbean Hispanic dataset was included, five SNPs (rs16945088, rs9931164, rs17219084, rs11075996, rs11075997) were significantly associated with AD. Adjustment for *APOE* genotype did not change these results, and there was no interactive effect of SNPs in *FTO* and APOE genotype on AD risk in either dataset.

**Table 2:** Results from single marker association analyses.

NIALOAD (AD)									
CHR	SNP	BP	A1	F_A	F_U	A2	P	OR	SE
16	rs6499640	52327178	G	0.41	0.39	A	0.05	1.14	0.07
16	rs10852521	52362466	T	0.51	0.48	C	0.09	1.11	0.06
16	rs16945088	52370025	G	0.08	0.09	A	0.09	0.82	0.11
16	rs8044769	52396636	T	0.49	0.47	C	0.09	1.11	0.07

Caribbean Hispanics (AD)									
CHR	SNP	BP	A1	F_A	F_U	A2	P	OR	SE
16	rs9931164	52,382,739	G	0.02	0.04	A	0.09	0.66	0.25
16	rs17219084	52,413,101	G	0.39	0.34	A	0.01	1.25	0.09
16	rs11075996	52,415,525	T	0.49	0.44	C	0.009	1.25	0.09
16	rs11075997	52,416,413	T	0.50	0.45	C	0.01	1.24	0.09

A1 = minor allele; A2 = wild type allele; p = p-value; OR = odds ratio, SE = standard error: F_A = frequency of minor allele in affecteds, F_U = frequency of minor allele in unaffecteds.

**Table 3:** Results from haplotype analyses.

NIALOAD						
SNPS	HAPLOTYPE	F_A	F_U	CHISQ	DF	P
rs6499646|rs1421090|rs17219084	TCA	0.04	0.17	3.93	1	0.04

Caribbean Hispanics						
SNPS	HAPLOTYPE	F_A	F_U	CHISQ	DF	P
rs12597786|rs7201850|rs9931164	CTG	0.02	0.04	2.80	1	0.09
rs7201850|rs9931164|rs9941349	TGT	0.02	0.04	2.73	1	0.09
rs9931164|rs9941349|rs7199182	GTA	0.02	0.04	2.73	1	0.09
rs8044769|rs6499646|rs1421090	TTC	0.06	0.08	4.51	1	0.03
rs6499646|rs1421090|rs17219084	TTG	0.24	0.19	6.26	1	0.01
rs6499646|rs1421090|rs17219084	TCA	0.08	0.12	6.33	1	0.01
rs1421090|rs17219084|rs11075996	TGT	0.32	0.26	8.10	1	0.004
rs1421090|rs17219084|rs11075996	CAC	0.11	0.15	4.86	1	0.02
rs17219084|rs11075996|rs11075997	GTT	0.38	0.33	5.82	1	0.01
rs17219084|rs11075996|rs11075997	ACC	0.50	0.55	6.74	1	0.009

F_A = frequency of minor allele in affecteds, F_U = frequency of minor allele in unaffecteds; CHISQ = χ2 test statistic; DF = degrees of freedom; p = p-value.

**Table 4:** Results from Metaanalyses.

Metaanalysis NIALOAD+ADNI									
CHR	SNP	BP	A1	A2	P	P(R)	OR	OR(R)	Q
16	rs6499640	52327178	G	A	0.05	0.05401	1.1148	1.1148	0.6
16	rs16945088	52370025	G	A	0.006	0.01041	0.7685	0.7649	0.3
16	rs6499646	52401034	C	T	0.03	0.1122	0.815	0.7918	0.2

Metaanalysis NIALOAD+ADNI+Caribbean Hispanics									
CHR	SNP	BP	A1	A2	P	P(R)	OR	OR(R)	Q
16	rs16945088	52370025	G	A	0.01	0.03477	0.8366	0.8268	0.2
16	rs9931164	52382739	G	A	0.03	0.02935	0.7198	0.7198	0.8
16	rs17219084	52413101	G	A	0.03	0.08434	1.1102	1.1284	0.2
16	rs11075996	52415525	T	C	0.01	0.07157	1.118	1.1376	0.1
16	rs11075997	52416413	T	C	0.02	0.07655	1.1117	1.1278	0.2

A1 = minor allele; A2 = wild type allele; P = Fixed-effects meta-analysis p-value; P(R) = random-effects meta-analysis p-value; OR = Fixed-effects meta-analysis odds ratio; OR(R) = random-effects meta-analysis odds ratio; Q = p-value for Cochrane's Q statistic.

### Microarray gene expression analyses

**Figure 3:**
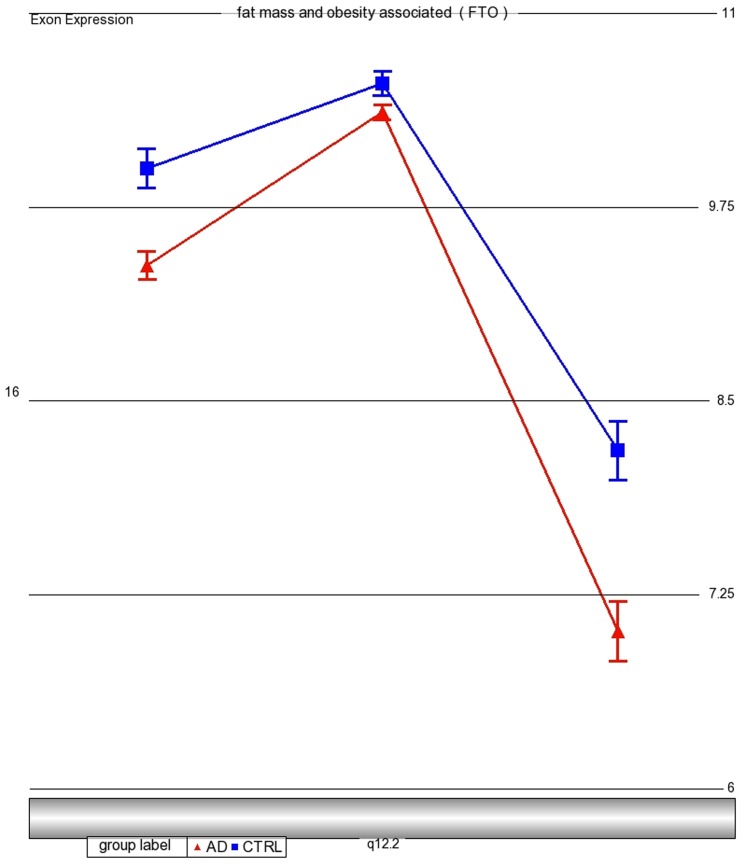
View of *FTO* exon expression profile in 19 AD (red triangles) and ten control (blue squares) amygdala tissue. Each triangle dot represents least squares mean expression of an exon in AD tissue; each square dot represents least squares mean expression of an exon in control tissue. The mean gene expression intensity of AD vs. Controls was 8.91±0.36 vs 9.57±0.32 (p = 2.18×10−5) across all exons.

While there were no differences in expression levels in tissue derived from the cerebellum or occipital lobe, microarray expression analyses of the amygdala tissue from the 19 AD and 10 control brains showed significantly lower expression of *FTO* in AD brains compared to control brains (mean gene expression intensity: 8.91±0.36 vs 9.57±0.23, p = 2.1E-5; Figure 3). These findings were validated by comparison with publicly available gene expression results (188 AD cases, 176 controls: mean expression intensity 594.92±148.2 vs. 680.23±139.65, p<0.0001, http://labs.med.miami.edu/myers/) [44]. In this publicly available dataset, logistic regression analyses relating SNPs in *FTO* with *FTO* gene expression levels suggested that the A allele of rs9972717 residing in intron 2 may be positively associated with *FTO* expression levels (β = 44.4, SE 14.61, nominal p = 0.002, FDR p-value: 0.05, Table S2), further providing support for a functional role of this genetic region.

## Discussion

The findings reported here confirm the association between genetic variation in Intron 1, Exon 2 or Intron 2 in the *FTO* gene and AD. Several SNPs in this region of the gene were associated with AD in Caucasians of European ancestry as well as in Caribbean Hispanics. In addition, *FTO* was significantly lower expressed in AD cases compared to controls in two independent datasets and there was an effect of genetic variation in intron 2 on *FTO* expression levels.

These results are consistent with epidemiological studies relating obesity measures with AD [4], [5], [8], [45]. In addition, they are consistent with the findings of genetic associations between variation in *FTO* and obesity measures with brain volume [19], verbal fluency [20] and the previous study reporting an association of the rs9939609 SNP with AD [21]. Of note, consistent with the previous reports, several of the disease-associated SNPs are located in the 47 kb LD block that spans Intron1, Exon2 and Intron2, and are in tight LD with the SNPs previously reported to be associated with obesity, obesity-related traits, brain volume, verbal fluency and AD. Other SNPs are located downstream in Intron 2 and have not been reported before. The occurrence of pathogenic mutations across multiple domains of disease genes (allelic heterogeneity) and the absence of these variants in some datasets or ethnic groups (locus heterogeneity) are frequently observed in both monogenic and complex traits. As expected, the effect sizes of associated SNPs were modest (OR 1.1–1.2). This is consistent with the notion of a complex disease and all recently detected novel AD susceptibility loci [46], [47], [48], [49], [50] and may explain why the *FTO* locus has not been reported by the recent large GWAS studies which may have been underpowered when correcting for total the number of genome-wide performed tests.

There are several potential mechanisms that could link obesity and AD. Obesity is a risk factor for hyperinsulinemia and T2D [51] and both are risk factors for AD [52]. Obesity is also related to other vascular risk factors such as hypertension and dyslipidemia, heart disease, and stroke, which have also been reported to be associated with AD in isolation and in aggregate [53]. Finally, obesity is also related to the production of adipokines and cytokines [54], which are correlates of hyperinsulinemia and T2D although their independent role in LOAD is less clear.

It has to be noted that the SNPs assessed were derived from the available genome-wide screening in all datasets. Thus, they do not cover the complete genetic variation in Intron 1, Exon2 and Intron 2 and it is possible that there are additional disease-associated markers that have not been genotyped. It is also possible that there are disease-associated variants in other regions of the gene, or that we lacked power to detect additional disease-associated markers with lower allele frequencies or effect sizes.

Taken together, our results suggest that FTO is causally involved in AD. Future studies should include comprehensive sequencing analysis to identify the specific causative sequence variants underlying the detected associations.

## Supporting Information

Table S1 Platforms used for *APOE* genotyping.
(DOCX)

Table S2 Effect of genetic variation in FTO on FTO expression levels.
(DOCX)
