# Supplementary material for: Genetic Variants in the Fat and Obesity Associated (FTO) Gene and Risk of Alzheimer's Disease
Source: PLoS One. 2012 Dec 12;7(12):e50354. doi: 10.1371/journal.pone.0050354 (PMC3520931; doi:10.1371/journal.pone.0050354)
Supplement: Table S1 — Platforms used for APOE genotyping. (DOCX) [file pone.0050354.s001.docx]

| **Dataset** | ***APOE* genotyping** |
| --- | --- |
| **NIA-LOAD/ADGC** | Prevention Genetics based on allelic combinations of SNPs rs7412 and rs429358 |
| **CARIBBEAN HISPANIC** | determined using the method of Hixson and Vernier[^39^](#_ENREF_39) |
