# Supplementary material for: Genetic Variants in the Fat and Obesity Associated (FTO) Gene and Risk of Alzheimer's Disease
Source: PLoS One. 2012 Dec 12;7(12):e50354. doi: 10.1371/journal.pone.0050354 (PMC3520931; doi:10.1371/journal.pone.0050354)
Supplement: Table S2 — Effect of genetic variation in FTO on FTO expression levels. (DOCX) [file pone.0050354.s002.docx]

| **CHR** | **SNP** | **BP** | **N** | **BETA** | **SE** | **P** | **FDR_BH** |
| --- | --- | --- | --- | --- | --- | --- | --- |
| 16 | rs1108102 | 52,347,009 | 350 | -49.2 | 56.9 | 0.39 | 0.78 |
| 16 | rs1861869 | 52,347,682 | 358 | -0.4 | 10.9 | 0.97 | 0.97 |
| 16 | rs1861868 | 52,347,903 | 355 | 2.4 | 10.9 | 0.83 | 0.87 |
| 16 | rs9940700 | 52,352,910 | 338 | 20.9 | 14.7 | 0.16 | 0.62 |
| 16 | rs9939973 | 52,358,069 | 364 | 4.9 | 11.5 | 0.67 | 0.84 |
| 16 | rs9940128 | 52,358,255 | 362 | 4.8 | 11.5 | 0.68 | 0.84 |
| 16 | rs9922047 | 52,363,781 | 362 | -13.1 | 11.1 | 0.24 | 0.70 |
| 16 | rs16952522 | 52,364,999 | 362 | 52.9 | 26.5 | 0.05 | 0.47 |
| 16 | rs17817288 | 52,365,265 | 362 | -19.2 | 11.2 | 0.09 | 0.59 |
| 16 | rs1477196 | 52,365,759 | 358 | -17.4 | 11.7 | 0.14 | 0.62 |
| 16 | rs1121980 | 52,366,748 | 361 | 5.9 | 11.5 | 0.61 | 0.84 |
| 16 | rs7193144 | 52,368,187 | 355 | 4.8 | 11.9 | 0.69 | 0.84 |
| 16 | rs16945088 | 52,370,025 | 343 | 14.0 | 18.2 | 0.44 | 0.80 |
| 16 | rs9926289 | 52,378,004 | 351 | 2.5 | 11.7 | 0.83 | 0.87 |
| 16 | rs9939609 | 52,378,028 | 359 | 7.6 | 11.9 | 0.52 | 0.84 |
| 16 | rs9930506 | 52,387,966 | 353 | 10.3 | 11.4 | 0.37 | 0.78 |
| 16 | rs11075994 | 52,407,580 | 362 | -4.5 | 12.3 | 0.72 | 0.84 |
| 16 | rs1421090 | 52,407,671 | 364 | 12.2 | 13.0 | 0.35 | 0.78 |
| **16** | **rs9972717** | **52,408,805** | **360** | **44.4** | **14.6** | **0.002** | **0.05** |
| 16 | rs10852522 | 52,416,278 | 363 | 12.9 | 11.1 | 0.25 | 0.70 |
